# Inferring common cognitive mechanisms from brain blood-flow lateralization data: a new methodology for fTCD analysis

**DOI:** 10.3389/fpsyg.2014.00552

**Published:** 2014-06-16

**Authors:** Georg F. Meyer, Amy Spray, Jo E. Fairlie, Natalie T. Uomini

**Affiliations:** ^1^Department of Psychological Sciences, University of LiverpoolLiverpool, UK; ^2^School of Psychology, University of LiverpoolLiverpool, UK; ^3^Department of Archaeology, Classics and Egyptology, University of LiverpoolLiverpool, UK

**Keywords:** Tower of London, music, temporal patterns, hemodynamics, middle cerebral artery, cued word generation, language, lateralization

## Abstract

Current neuroimaging techniques with high spatial resolution constrain participant motion so that many natural tasks cannot be carried out. The aim of this paper is to show how a time-locked correlation-analysis of cerebral blood flow velocity (CBFV) lateralization data, obtained with functional TransCranial Doppler (fTCD) ultrasound, can be used to infer cerebral activation patterns across tasks. In a first experiment we demonstrate that the proposed analysis method results in data that are comparable with the standard Lateralization Index (LI) for within-task comparisons of CBFV patterns, recorded during cued word generation (CWG) at two difficulty levels. In the main experiment we demonstrate that the proposed analysis method shows correlated blood-flow patterns for two different cognitive tasks that are known to draw on common brain areas, CWG, and Music Synthesis. We show that CBFV patterns for Music and CWG are correlated only for participants with prior musical training. CBFV patterns for tasks that draw on distinct brain areas, the Tower of London and CWG, are not correlated. The proposed methodology extends conventional fTCD analysis by including temporal information in the analysis of cerebral blood-flow patterns to provide a robust, non-invasive method to infer whether common brain areas are used in different cognitive tasks. It complements conventional high resolution imaging techniques.

## Introduction

Functional TransCranial Doppler (fTCD) ultrasound scanning is a well established technique for the robust measurement of cerebral lateralization during cognitive tasks (Knecht et al., 1998a,b; Deppe et al., 2004). It offers reliable measurements of the precise time course of cerebral blood flow changes, using portable equipment that is not susceptible to motion artefacts (e.g., Uomini and Meyer, 2013), but provides very limited spatial information.

Complementary to this, fMRI provides very high resolution imaging data that can be used to map brain areas (Newman et al., 2003; Jansen et al., 2006; Price, 2010; Meyer et al., 2011), network connectivity (Basser and Jones, 2002; Beer et al., 2013), and to decode representational content using techniques such as multi voxel pattern association (Norman et al., 2006) during specific tasks. While there is no question that fMRI is the benchmark experimental technique in cognitive neuroscience, it has a number of drawbacks, chiefly its sensitivity to participant motion (Seto et al., 2001), which requires participants to lie motionless while executing tasks.

Tasks that require participants to produce actions that can cause head movements inside the scanner are therefore a particular challenge for fMRI. FTCD has been shown to provide highly replicable measurements while participants perform actions that range from simple actions, such as elbow flexion/extension (Salinet et al., 2012), or speaking (Bishop et al., 2009) in laboratory environments, to highly energetic stone tool making (Uomini and Meyer, 2013) or driving a car in a driving simulator (Lust et al., 2011).

While fTCD has very poor spatial resolution, it provides robust temporal cerebral blood-flow signatures. Temporal data are not the focus of conventional fTCD analysis where peak blood-flow lateralization measures are reported. We propose an extension of current fTCD analysis methods that explicitly takes the temporal dynamics into account to compare blood-flow signatures for different cognitive tasks. We argue that tasks that draw on common brain areas should result in correlated activation patterns, while tasks that draw on different brain areas result in uncorrelated patterns. While the proposed analysis method is no alternative to fMRI, it provides complementary data that can be used in situations where conventional high resolution neuroimaging would not be possible, such as in natural environments, during active motion, or for participants who would be ineligible for scanning. Blood-flow signatures for tasks or participant groups that would not be suitable for conventional scanning can be directly compared with appropriate benchmark data to infer whether common processing networks are used.

### fTCD overview

**Figure 1:**
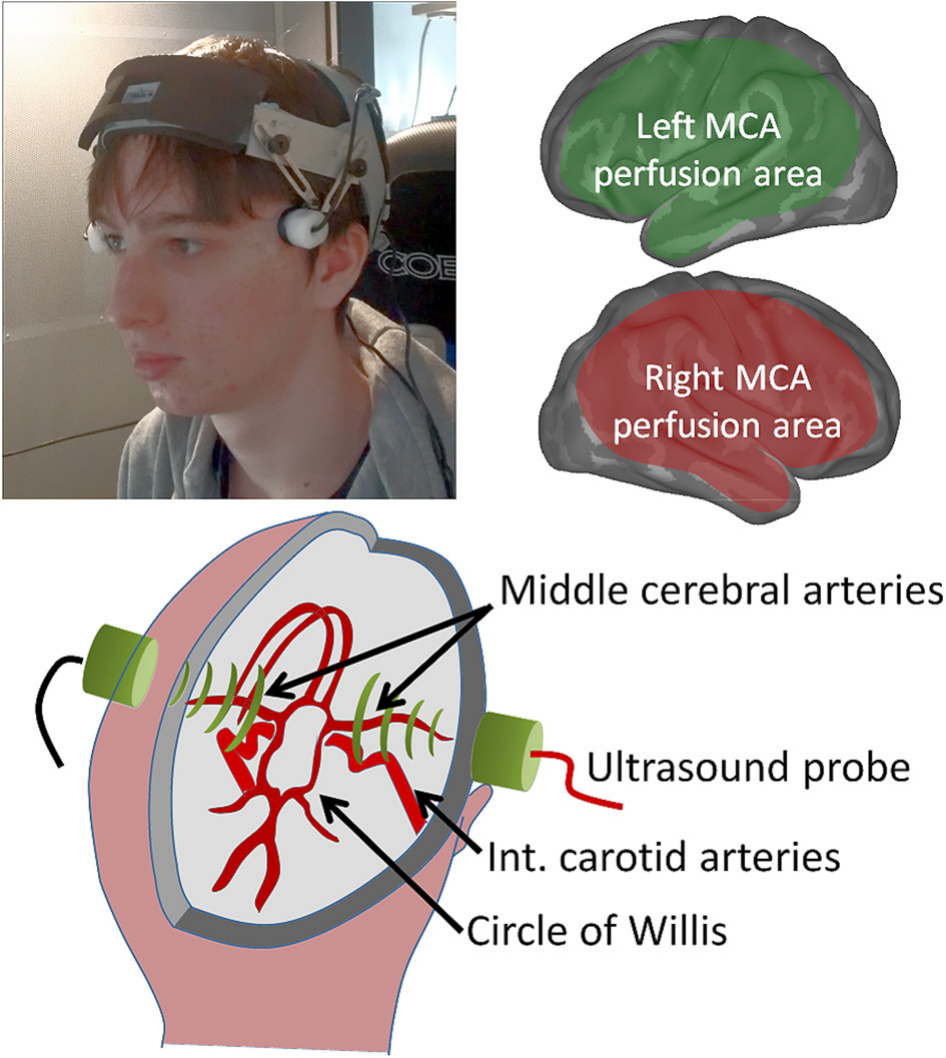
**Bilateral cerebral blood-flow is recorded using two small head-mounted probes (top left) that are relatively insensitive to participant motion**. The middle cerebral arteries, insonated at a depth of ca 5 cm as they emerge from the circle of Willis (lower schematic) supply extensive regions of the cerebral cortex (top right schematic), but this excludes frontal and sagittal areas, which are supplied by the anterior cerebral artery, and occipital and inferior temporal brain areas supplied by the posterior cerebral artery.

FTCD measures blood flow velocity and volume changes in the major arteries supplying the brain (Deppe et al., 1997, 2000; Duschek and Schandry, 2003; Bishop et al., 2009) using two small head-mounted sensors, Figure 1. The technique has been used extensively for language lateralization studies since 1998, providing well documented and highly replicable baseline data (Knecht et al., 1998a; Stroobant and Vingerhoets, 2001; Bishop et al., 2009; Illingworth and Bishop, 2009; Groen et al., 2012).

### Paradigm

Cerebral lateralization is measured by computing the change in bilateral blood flow velocity in the major arteries supplying the brain during the execution of specific cognitive tasks. The changes are measured by comparing multiple cycles of alternating target and rest periods that each last around 30 s.

Cued word generation (CWG), a task where participants are asked to silently think of as many words as possible starting with a given letter, has been used extensively in language lateralization studies (Knecht et al., 1998a; Deppe et al., 2004). This task is used as one of the cognitive tasks in all experiments reported here because it has a wealth of comparison data from fTCD and other imaging methodologies.

### Lateralization index

The fTCD lateralization index (LI) was developed to assess language lateralization in a clinical context (Knecht et al., 1998a,b). Doppler ultrasound is used to measure blood flow velocity in a pair of left and right cerebral arteries, typically the middle cerebral arteries (MCAs) supplying the brain. Relative blood-flow velocity changes compared to a baseline provide a robust estimate of the change in blood-flow volume. The LI is the difference in bilateral cerebral blood flow volume (CBFV) changes, *dV(t)*, during task execution relative to a baseline (Equation 1, adapted from Knecht et al., 1998a).

(1) $$\Delta V(t)=dV_{left}(t)-dV_{right}(t),$$

where

$$dV(t)=100(V(t)-V_{b})/V_{b}$$

is the CBFV change relative to the mean baseline blood flow velocity (*V*_b_), typically recorded over the 5 s preceding the target condition onset. The lateralization time course (Δ*V*(*t*)) is a continuous function that changes during task execution and is specific for each individual.

The *LI*, represents the maximum absolute lateralization value, averaged over an integration interval, within the activation interval (Equation 2 adapted from Knecht et al., 1998a):

(2) $$LI=\frac{1}{t_{int}}\int_{t_{\max}-0.5t_{int}}^{t_{\max}+0.5t_{int}}\Delta V(t)dt$$

A time period of *t_int_* = 2 s is typically chosen as the integration interval. A positive value of the LI indicates left hemispheric processing dominance while negative values represent right hemisphere dominance. Our proposed analysis method builds on this well-established technique.

### Replicability

A number of studies have shown that fTCD provides highly replicable data that match other measures of cerebral activation. Cerebral blood flow lateralization data obtained with fTCD match alternative measures, such as the relative distribution of fMRI voxel counts for cued word generation (CWG) (Deppe et al., 2000; Somers et al., 2011) and spatial attention tasks (Jansen et al., 2004, 2006). Sabri et al. (2003) showed a very high correlation between simultaneously recorded PET and fTCD lateralization data in a (n-back) working memory task. Language lateralization measured with fTCD also predicts the effect of unilateral disruption of language functions via either the intracarotid sodium amobarbital procedure (Wada test) (Knecht et al., 1998b) or repetitive Transcranial Magnetic Stimulation (rTMS) (Flöel et al., 2000).

If two cognitive tasks draw on common brain areas, which will share common haemodynamics, then one would expect highly correlated responses across a pool of participants. Bishop et al. (2009) compared the LIs obtained with the CWG task with those measured for two other language tasks that rely more on syntactic processing. They show that LIs for the CWG task are highly correlated for all three cognitive tasks, as would be expected for tasks that draw on substantially overlapping cortical networks.

It could, of course, be argued that a change from rest to any cognitive task leads to common increases in cortical activation or common attentional processes, so that correlated LIs might be expected for any pair of tasks. This is not the case. A number of studies show that visuo-spatial tasks, which draw on different brain areas than language tasks, lead to LIs that are *not* correlated with the standard CWG task: Rosch et al. (2012) tested visuo-spatial attention, Whitehouse et al. (2009), Whitehouse and Bishop (2009) used a visual memory task, while Lust et al. (2011) tested participants in a driving simulator. None of these studies found a correlation with CWG, showing that common, non-task specific processes, for example attentional modulation, are not a trivial explanation for correlated LI patterns.

Rosch et al. (2012) showed that visuospatial laterality measures were highly intercorrelated and unaffected by task difficulty, while Badcock et al. (2012) showed that for the standard CWG and an auditory naming task, performance, and reaction time measures co-varied with task difficulty while lateralization measures were not significantly different. This means that specific task demands, a difficult to control confound when two different cognitive tasks are compared, are not a sufficient explanation for the absence of correlated LI values.

### Correlation analysis

The fundamental question we address in this paper is how individual CBFV lateralization traces can serve as the basis for inferences about common underlying brain areas that are used for *different* cognitive tasks. We argue that correlated haemodynamics provide this indication. While the LI is an appropriate measure to quantify hemispheric dominance for a given task, we argue that a comparison of peak values, the basis of the LI, is not the most appropriate measure for cross-task comparisons.

FMRI studies consistently show that, while one hemisphere is often dominant (e.g., language is typically left dominant; visuospatial processing is often right dominant), both hemispheres significantly contribute to most cognitive tasks (Bradshaw and Nettleton, 1982; Stroobant and Vingerhoets, 2001; Hickok and Poeppel, 2004; Whitehouse et al., 2009; Meyer et al., 2011; Somers et al., 2011; Groen et al., 2012; Rosch et al., 2012; Wuerger et al., 2012). A positive (left) LI for language, for example, should therefore not be interpreted as showing that language exclusively uses the left hemisphere. Instead it shows that a proportion of the underlying cognitive processes are left *dominant*.

This observation has two important implications. The first is that common overall lateralization of cerebral blood flow patterns during two tasks is not sufficient evidence for common underlying processing. It is entirely plausible that two tasks, which draw on non-overlapping brain areas, are dominant in the same hemisphere. In this case we would expect to see common overall lateralization, but not correlated CBFV patterns because each brain area has its own haemodynamics. Secondly, properly considering the LI measures as a relative dominance of cerebral activation also means that two cognitive processes can result in opposite lateralization indices for the same participants, *even if* they share significant processing. Music (right dominant) and language (left) are two well documented examples (reviews: EEG and fMRI data: Koelsch, 2012; PET data: Evers et al., 1999; Brown et al., 2006). Here both tasks draw on extensive, shared, bilateral networks but language—on average—activates more left lateralized brain areas while music draws on slightly right dominant networks. Experiment two will demonstrate this.

The analysis proposed here is based on the measured *degree* of lateralization *in a population of subjects* and follows existing cross-methodology (fMRI or PET correlated with fTCD: Deppe et al., 2000; Sabri et al., 2003) and cross-task (language vs. language/visuo-spatial/memory: Bishop et al., 2009; Whitehouse and Bishop, 2009; Whitehouse et al., 2009; Rosch et al., 2012) comparisons of cerebral lateralization.

An important methodological difference to the conventional LI analysis derives from our argument that choosing a single maximum value (the LI) as the measure of lateralization is potentially misleading because important temporal information is lost.

The time course and peak lateralization of individual fTCD recordings varies significantly between individuals, but both are highly replicable within each individual. This means that these haemodynamic variations are caused by idiosyncratic differences in the activation of brain areas rather than “noise.” This is consistent with data reported in fMRI: despite the consistency of the spatially localized response patterns across subjects there is a marked, idiosyncratic variation in the timing and shape of BOLD responses across subjects (Schacter et al., 1997; Aguirre et al., 1998; Buckner et al., 2000). The source of this variability is presently unclear, but may be caused by differences in blood vessel density across regions (Lee et al., 1995), or by systematic processing delays in the underlying neuronal networks (Rosen et al., 1998).

If two tasks share common dominant brain areas, then we expect not only correlated peak lateralization values across participants, which provide the basis of the LI calculation, but we also expect lateralization *changes* to occur simultaneously for both tasks within the same participant. We therefore argue that for a principled analysis, an additional constraint should be imposed: to meaningfully compare time variant lateralization data, LI values should be correlated only within relatively narrow, synchronous analysis windows for the two tasks under consideration. We propose a moving average window of 5 s duration, which is in line with the temporal window in which BOLD responses in fMRI can be resolved (Glover, 1999; Jäncke et al., 1999).

The analysis method we propose therefore draws on the conventional LI calculation, but instead of estimating lateralization from single peak values, lateralization signatures from two tasks are compared by computing a running cross-correlation of the cerebral blood flow differences measured in successive 5 s analysis windows for a population of participants.

## General materials and methods

All experiments reported in this paper follow a similar experimental design and use the same recording equipment, methodology, and data analysis. This section details the aspects of experimental design and analysis that are common to all three experiments.

### Subjects

Participants were recruited by opportunity sampling. The majority were undergraduate students at the University of Liverpool, who were given course credits for their participation. All were healthy and without a history of neurological disorder. All had normal or corrected to normal vision and reported no hearing problems.

### Ethics statement

The experiments were approved by the University of Liverpool ethics committee (reference PSYC-1011-025—Georg Meyer—Action planning and cerebral blood flow lateralization). Written informed consent was acquired from all participants. The participant shown in Figure 1 gave written informed consent to the publication of his image.

### Apparatus and materials

A schematic diagram of the fTCD setup and a picture of the fTCD probes in use during an experiment are shown in Figure 1. Blood-flow changes are simultaneously measured in both MCAs at a depth of approximately 50 mm with a commercially available dual transcranial Doppler ultrasonography device (Multi-Dop T, DWL, Sipplingen, Germany). The two 2-MHz transducer probes were mounted on an Integra UltraLite headband (001270BIF, Integra LifeScience Corp, USA) and placed at the trans-temporal windows. The spectral envelope curves of the Doppler signals were recorded with a sample rate of 25 Hz.

### Experimental conditions

We compare relative MCA CBFV changes during two cognitive tasks in all experiments. In both experimental conditions, target intervals were alternated with control intervals. Following standard fTCD paradigms (Deppe et al., 1997; Knecht et al., 1998a, 2000) the target intervals were 25–35 s (average =30 s) in duration while the control conditions were 15–25 s (average = 20 s) long. Twenty target/control epochs were presented in each experimental block. Stimulus presentation was controlled by a personal computer running the ShowPics software (v. 3.1.0) which was interfaced using parallel port TTL signals to the analog input of the fTCD system to mark the start of each epoch.

The CWG task, used in all experiments reported here, is a standard language lateralization assessment task used in clinical settings (Knecht et al., 2000). Subjects were asked to silently generate words starting with a letter heard at the onset of the target interval. The same letter sequence was presented to all participants in experiment 2: *[H, L, O, N, C, P, Q, T, U, Z, K, J, D, U, R, S, B, A, W, I]*. For the control interval subjects were asked to rest silently. A beep and a spoken letter marked the onset of the target interval while an isolated beep indicated the start of the control interval. In contrast to many CWG paradigms, our participants were not required to report words verbally, so that CWG and rest blocks alternated in direct succession.

The same number (20) and timing of target and control intervals was used in all experiments. Each cognitive task, e.g., 20 trials of CWG, was carried out as a separate block lasting approximately 15 min. The order of blocks within each experiment was randomized to control for order effects.

### Data analysis

The recordings were integrated over the corresponding cardiac cycles, segmented into epochs and then averaged off-line using the AVERAGE V1.85 software (Deppe et al., 1997). Trials with physiologically implausible CBFV changes relative to baseline of ±30% were excluded from the analysis. Subjects with less than 80% “good” epochs in any one of the conditions were excluded from the data analysis to ensure data integrity. The raw blood flow data are integrated over cardiac cycles, so that the CBFV signal is characterized by successive constant segments with sudden (high frequency) transitions at the time when heartbeats are detected. The average responses were filtered off-line using a second order zero-phase lag Butterworth low-pass filter to remove these high frequency components. A cut-off frequency of 1 Hz, the Nyquist limit for sampling at relatively high heart rates of 120 bpm (2 Hz), was used to ensure that haemodynamic responses were retained. All CBFV changes are computed relative to a baseline that was the average of the 5 s period immediately preceding the target epoch onset. Group statistics were computed using purpose-designed MATLAB (The Mathworks, Natick, MA) scripts.

CBFV lateralization differences (LIs) are computed not at the maximum LI, but for each sample in the measurement time series, for the average CBFV difference in a *t_int_* = 5 s interval. The LI value is computed separately for each participant, *p*, and for each of the two conditions, *c*, to be compared.

(3) $$LI(p,c,t)=\frac{1}{t_{int}}\int_{t}^{t+t_{int}}\Delta V(t)dt$$

Two series, one for each of two cognitive tasks (*c*_1_ and c_2_) that were executed by the same participants (*P* = [*p*_1._. *p_N_*]) can then be correlated to obtain a running similarity measure

$$R_{c1,c2}(t)=r\left(LI_{P,c1}(t),LI_{P,c2}(t)\right)$$

where *r(x,y)* is the Pearson product-moment correlation coefficient.

## Validation experiments

The aim of this paper is to show that the proposed methodology enables a principled comparison of CBFV change data for a population of participants within and across tasks.

We make the case that the time course, and with it the peak value and latency, of the haemodynamic response varies systematically with the specific brain areas each individual uses to perform cognitive tasks, and their blood flow patterns. These idiosyncratic responses, however, are highly replicable. Tasks that draw on the same or substantially overlapping brain areas will therefore result in similar cerebral blood-flow signatures. We argue that a time-locked, moving cross-correlation of CBFV differences across a participant pool is an appropriate analysis method.

In experiment 1 we show that the proposed analysis provides results that are comparable to those obtained with the conventional LI calculation. CBFV signatures for the CWG task stay highly correlated throughout the task interval when the task difficulty is manipulated.

In a second experiment we correlate CWG lateralization signatures with those for a music synthesis task and an abstract problem solving task, the Tower of London (ToL) problem. We expect the CBFV signatures for the language and music tasks, which have previously been shown to draw on overlapping brain areas, to be highly correlated. Conversely, we expect the CBFV signatures for the CWG and the ToL tasks to be uncorrelated because both tasks have previously been shown to draw on different brain areas.

### Experiment 1: the effect of task difficulty on lateralization measures

The Lateralization Index (LI) for the CWG task measured with fTCD has been shown to be highly replicable (Knecht et al., 1998a,b; Deppe et al., 2000; Flöel et al., 2001; Jansen et al., 2004). Rosch et al. (2012) argue that LIs obtained for lateralized visual attention are not influenced by task difficulty, while Schuepbach et al. (2007) provide evidence that early modulation of cerebral blood flow is correlated with performance and thereby task difficulty. The two claims are not contradictory because the LI measures the peak cerebral blood flow lateralization over an extended interval while Schuepbach et al. (2012) performed a much more detailed analysis of blood flow patterns during early stages of the responses where mean lateralization values typically lie below the peak values used for the LI calculation.

The cued word paradigm requires participants to recall words starting with a letter chosen from a random letter sequence. Some letters, for example C, R, S, and T, are much more common at the beginning of words than others (V, X, Y, Z) so that the choice of letters can be used to manipulate CWG task difficulty.

To create two lists of CWG starting letters (easy and hard), a group of 13 participants (mean age = 20.7 years, range = 20–26) was asked to loudly generate as many words starting with each cue letter in the alphabet as possible. The average number of words recalled for each 30 s period was counted and used to split the letters into “easy” letters (words starting with [P, B, L, H, W, E, F, C, T, O, V, R, M, G] average = 6.24 words/30 s, *SD* = 0.41 words generated) and “hard” letters (words starting with [Z, X, K, I, Q, Y, J, A, S, U, D, N], avg = 4.58 words/30 s, *SD* = 1.03 words generated). A paired *t*-test confirmed that these two sets led to significant performance differences [*t*_(12)_ = 8.971, *p* < 0.001].

In the fTCD experiment, CBFV changes in the MCAs were measured in 20 participants (mean age = 21.3 years, range = 18–38) using the standard procedure outline above. In each of the conditions participants were asked to silently generate as many words as possible starting with the cue letter.

**Figure 2:**
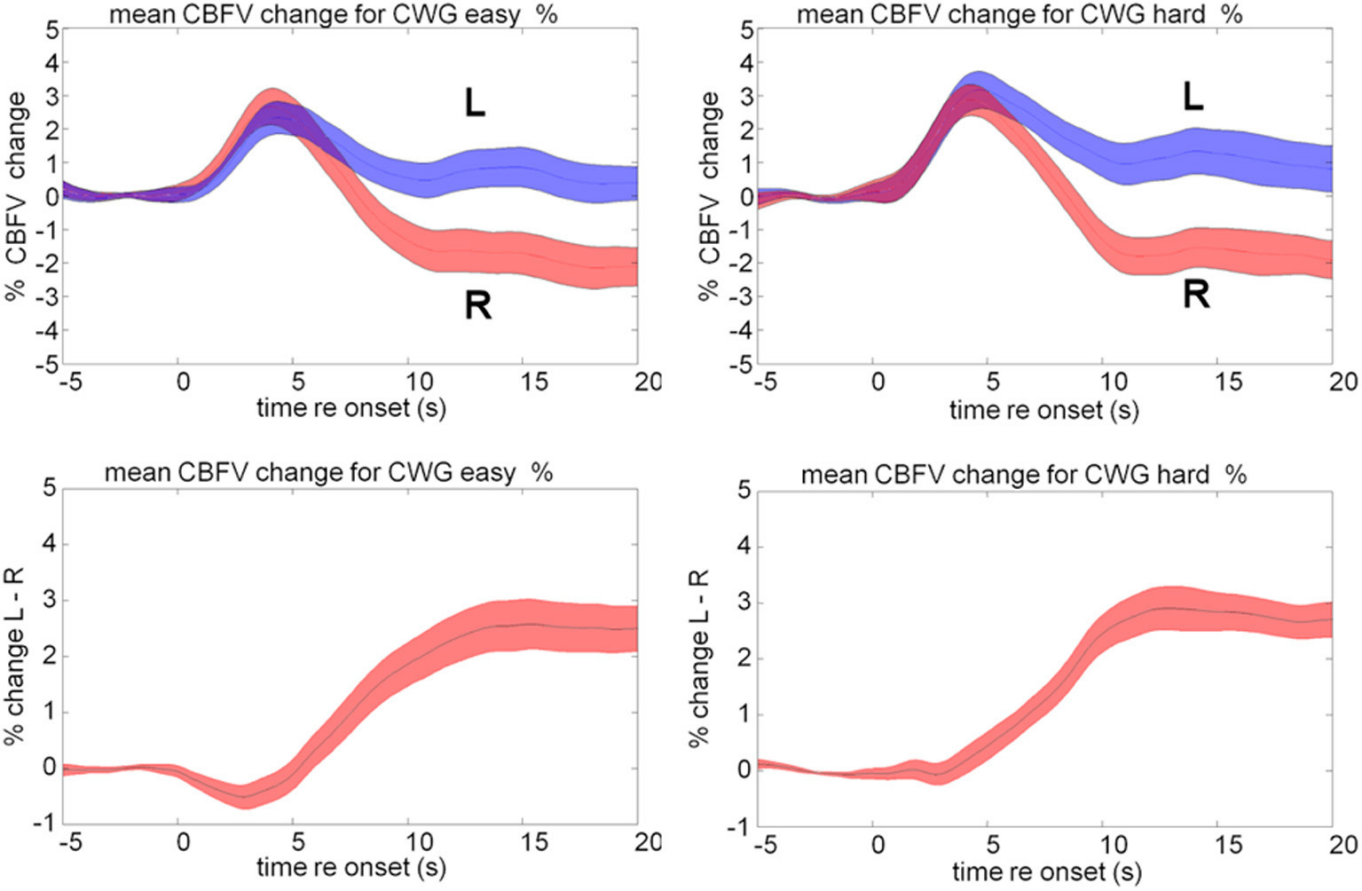
**Mean cerebral blood flow velocity (CBFV) change (%) in the left (blue) and right (red) middle cerebral arteries (top panels) during the hard and easy cued word generation tasks**. The bottom panel shows the difference between left and right in CBFV changes.

The average cerebral blood flow data for our group of 20 participants, Figure 2, shows a typical pattern for the CWG task. Activity in both MCAs rapidly increases over the initial 5 s of the CWG task condition. After the initial increase, the CBFV patterns in the two hemispheres diverge. The graphs show the mean CBFV change (Figure 2, top) and the difference between the left (L) and right (R) MCA CBFV. The standard error across all participants is shown as the colored bands around each line. The time course and magnitude of the mean data are consistent with previously reported data for the same task.

**Figure 3:**
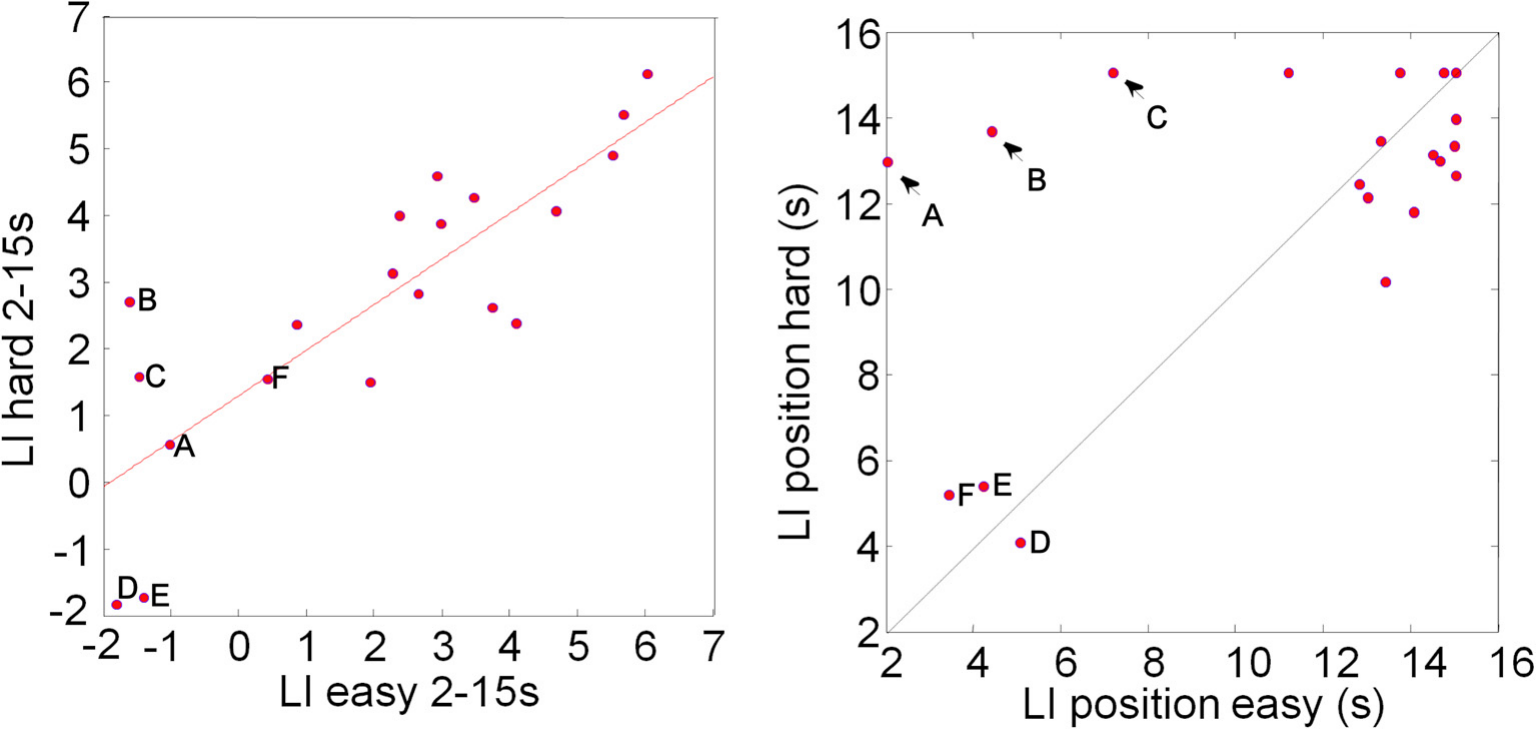
**Plot of Lateralization Indices (LI) computed for each participant in the easy and hard condition (left)**. The data show highly correlated (*r* = 0.83, *n* = 20, *p* = < 0.0001) LIs. The LIs are computed by averaging the CBFV difference in a window, here 2 s, around the absolute peak value in the analysis window, here 2–15 s post task onset. This means that, when LIs for two conditions or tasks are compared, the peak values do not have to coincide. The right hand graph shows the position of the windows that are the basis for the LI analysis: in the majority of cases LIs derive from similar positions between 10 and 15 s after task onset; for three individuals (A–C) peak LI values were found toward the end of the hard task conditions, but at the beginning of the “easy” conditions (arrows in right plot); for another three participants LIs were computed for relatively early responses (around 4 s) in both tasks. We argue that for a meaningful correlation analysis, matching time windows should be considered.

There are a number of ways to compare lateralization data across tasks or conditions. Rosch et al. (2012) directly compared the peak LI within the same participants. A paired *t*-test of the LI data plotted in Figure 3 shows no significant difference between the easy and hard condition (mean LI value easy = 2.28%, *SD* = 2.60%; mean LI value hard = 2.85%, *SD* = 2.15%; *t* = −0.84, *df* = 38, *p* = 0.4), consistent with data reported by Rosch et al. (2012) for a visual attention task. The LI values (Figure 3, left panel) are highly correlated (*r* = 0.83, *n* = 20, *p* = < 0.0001). It is, however, worth remembering that the LI is the maximum CBFV change difference during the task execution, irrespective of where this maximum occurred. The graph on the right of Figure 3 shows not the LI values, but the time (in seconds after task onset) where the peak value occurred: the majority of LI values (14/20) come from comparable positions toward the end of the task interval as expected, but for three participants the peak lateralization occurred quite early (<6 s after task onset) in both conditions. For another three participants (A–C, marked by arrows) the LIs were taken early during task execution in the easy condition, but late in the hard condition. We contend that this is not a meaningful comparison.

Duschek et al. (2008) investigated the relationship between rapid cerebral haemodynamic modulation and attentional performance. They demonstrated that blood flow relatively early in the stimulus interval (2–3 s after onset) was correlated with performance. This was not the case for the very early and late components of the response.

Our own data (Figure 2) show that the mean CBFV in the right MCA is not affected by a modulation of the task difficulty while the left MCA response differs during the early part of the response. In the easy condition, the left MCA response is slightly smaller and later than the right MCA response causing a dip reflecting a slight right lateralization bias on average that is visible between 0 and 5 s. In the hard condition this dip is not visible in the LI data. A direct pairwise comparison of the data shows a significant difference between the lateralization traces recorded during easy and hard CWG tasks between 2 and 3 s after task onset [mean CBFV difference easy = −0.16%, *SD* = 1.09; hard = + 0.32%, *SD* = 1.1; *t*_(38)_ = 1.71, *p* = 0.048]. This finding is consistent with Duschek et al. (2008) and in our view strengthens the case for a careful consideration of the temporal aspects of the haemodynamic response.

**Figure 4:**
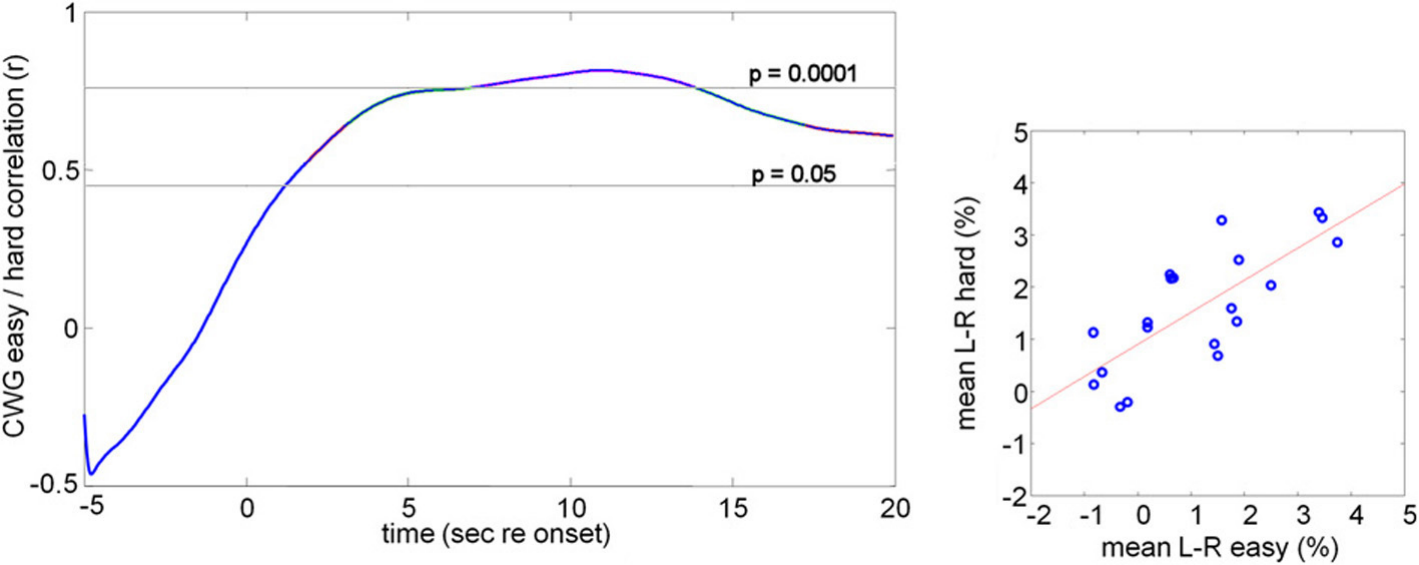
**Correlation of mean lateralization data**. The right hand graph shows the mean CBFV difference within 2–15 s after task onset for all participants. The data for the easy and hard conditions are highly correlated (*r* = 0.76, *n* = 20, *p* = 0.0001). This analysis assumes constant lateralization patterns over the entire duration of the analysis window. An alternative analysis is to compute the correlation between two conditions or tasks using a moving window of limited duration, here 5 s. The correlation coefficient is plotted against the start position of the moving analysis window (left hand graph). For this comparison we see a peak correlation of *r* = 0.81 (*p* < 0.0001) at 10 s after task onset.

Figure 4 shows the result of the correlation analysis we propose for comparing CBFV changes across tasks or task difficulty. Instead of correlating the (peak) LI values, we correlate the average CBFV difference within a succession of moving 5 s windows. The correlation could be computed at an a-priori defined small number of key comparison points, such as key points in the response or could be sampled continuously. Here we show continuous 5 s windows with start times between 5 s before the task onset (−5 s) and 20 s after the task onset.

The left hand graph shows correlation values, computed for a succession of Δ*V*(*t*) values averaged over a succession of 5 s long analysis windows with the indicated start times. For the two conditions the data are uncorrelated before the task onset, but during task execution highly correlated lateralization patterns are seen, as would be expected since the underlying task, and therefore the corresponding blood flow signatures, are essentially the same.

The right hand panel in Figure 4 shows highly correlated average lateralization patterns across the two conditions computed over the entire task interval (2–18 s post onset, *r* = 0.76, *n* = 20, *p* = 0.0001). The fact that the average CBFV over this long analysis interval shows a stable correlation between the two tasks is consistent with the continuous correlation in short analysis windows shown in the left panel. The correlation values are comparable with those obtained on the basis of the peak lateralization and show that the selection of only the peak value is not an essential feature of the analysis.

#### Summary

The data show highly correlated cerebral blood flow lateralization patterns for the duration of the task for two experimental conditions that differed significantly in task difficulty. This correlation is seen in a conventional LI analysis, but it is also seen when correlating the CBFV differences averaged over the range between 2 and 15 s, and when a succession of short term correlations with moving start points within this time range are carried out. The correlation values in the moving window analysis are significant even for windows starting only 2 s after task onset where absolute blood-flow changes are still small. The proposed analysis, therefore, is consistent with alternative methods for within-task comparisons. Our results extend the analysis to incorporate timing information, which as Duschek et al. (2008) have shown provides relevant information.

### Experiment 2: inferring common processing networks from correlated cerebral blood flow lateralization data

The aim of this paper is to show that different cognitive tasks that draw on common brain structures result in correlated cerebral blood-flow lateralization signatures. To this end we compare CBFV data for the “ToL” task and a music synthesis task with the CWG task introduced in experiment 1.

We hypothesize that language and music tasks, which have previously been shown to invoke substantially overlapping networks, will result in correlated CBFV data, while language and general abstract problem solving, exemplified by the ToL task, draw on largely distinct processing networks and should therefore result in uncorrelated CBFV signatures.

**Figure 5:**
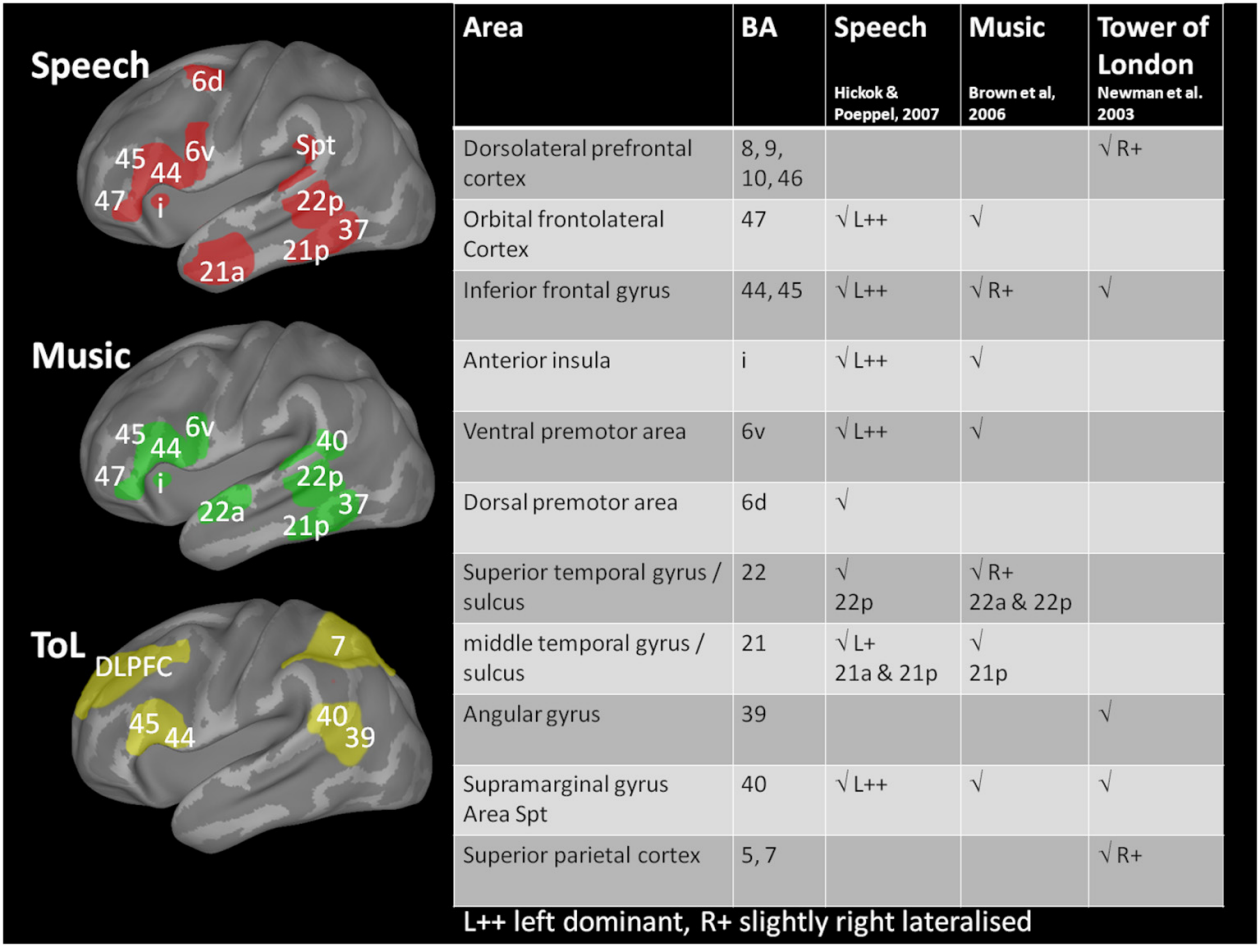
**Schematic diagram of brain activations associated with the three tasks**. Brodmann areas activated by language tasks are shown in red (from Hickok and Poeppel, 2007); very similar areas, but less left lateralized are involved in music perception and generation tasks (green, from Brown et al., 2006). Solving the Tower of London problem (yellow, from Newman et al., 2003) draws on different, frontolateral and parietal areas. The areas marked delineate Brodmann areas on an inflated brain representation (drawn with Caret, van Essen et al., 2001); primary sensory or motor areas are not shown. Activation is typically bilateral except where marked L++ for speech stimuli.

A summary of the major brain areas activated by language, music, and the ToL task is given in Figure 5. The music and ToL tasks are discussed in more detail in the following sections.

#### The tower of london

One of the standard tasks used to assess executive/planning processes and in particular visuo-spatial processing is the ToL task (Shallice, 1982; Baker et al., 1996). Behavioral data show that visuo-spatial abilities significantly predict TOL performance and that visuo-spatial, but not verbal, memory tasks interfere with ToL planning (Cheetham et al., 2012).

Frauenfelder et al. (2004) used fTCD to measure blood flow changes during the “Stockings of Cambridge” (SoC) task (Owen et al., 1990), a computer-screen based version of the ToL task, and found differences during planning and execution relative to a control condition where subjects were required to copy previously executed moves.

Neuroimaging studies (e.g., Newman et al., 2003, 2009) have shown that the ToL problem-solving task engages a large-scale, right dominant network of cortical regions, in particular the superior parietal and dorsolateral prefrontal cortex, but also inferior frontal gyrus and the inferior parietal cortex. This activation pattern shows overlap with the circuit invoked in CWG only in the inferior frontal gyrus (BA 45 46) (Chee et al., 1999; Buckner et al., 2000). We therefore expect the two tasks to cause lateralization to opposite sides of the brain and expect the lateralization data not to be correlated across participants.

#### The tower of london task

**Figure 6:**
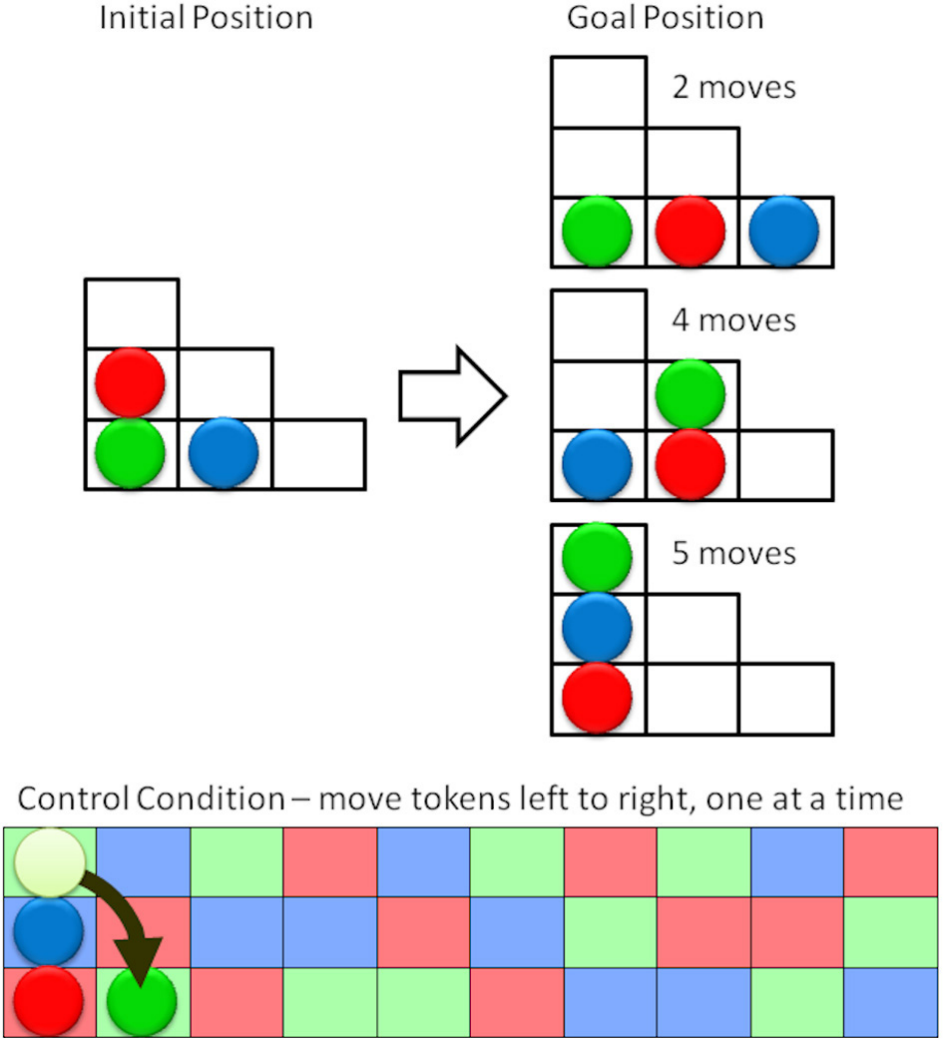
**Schematic representation of the Tower of London task (top) and the control condition (bottom)**. The aim is to move the three colored tokens from the initial position (left) to the goal position (right) in the minimum number of moves. Subjects were asked to first plan and then to execute their moves. In the control condition subjects were asked to move the tokens from left to right, following the color pattern. The next moves would be to move the blue token up and right, then the red token up and right, etc.

In the target condition participants were presented with a series of boards that had three colored tokens in the start configuration and a printed depiction of the target condition. Participants were asked to plan their moves before execution. As soon as a participant solved one puzzle, the next was presented, Figure 6 (top).

To isolate the planning from brain activity associated with moving the tokens on the board subjects were presented with a board containing a random sequence of the three token colors and were asked to move the tokens, one at a time, from their current position to the next matching square in the sequence, Figure 6 (bottom). As before 20 pairs of target and control conditions alternated within the experiment.

#### Music perception and generation

There are several arguments linking musical processing and language that range from a phylogenetic role in the evolution of language (review: Peretz and Zatorre, 2005), the ontogenesis of infant language (e.g., Trehub, 2003), to functional neuroimaging studies that show a close correspondence of brain areas involved in music and language processing (e.g., Koelsch et al., 2002; Hickok et al., 2003; Levitin and Menon, 2005; Patel, 2003; Koelsch, 2005; Brown et al., 2006; Fedorenko et al., 2009; Abrams et al., 2011).

If language and music draw on largely overlapping processing networks, then the haemodynamics observed during activation changes in similar tasks should be correlated across individual observers.

#### The music synthesis task

**Figure 7:**
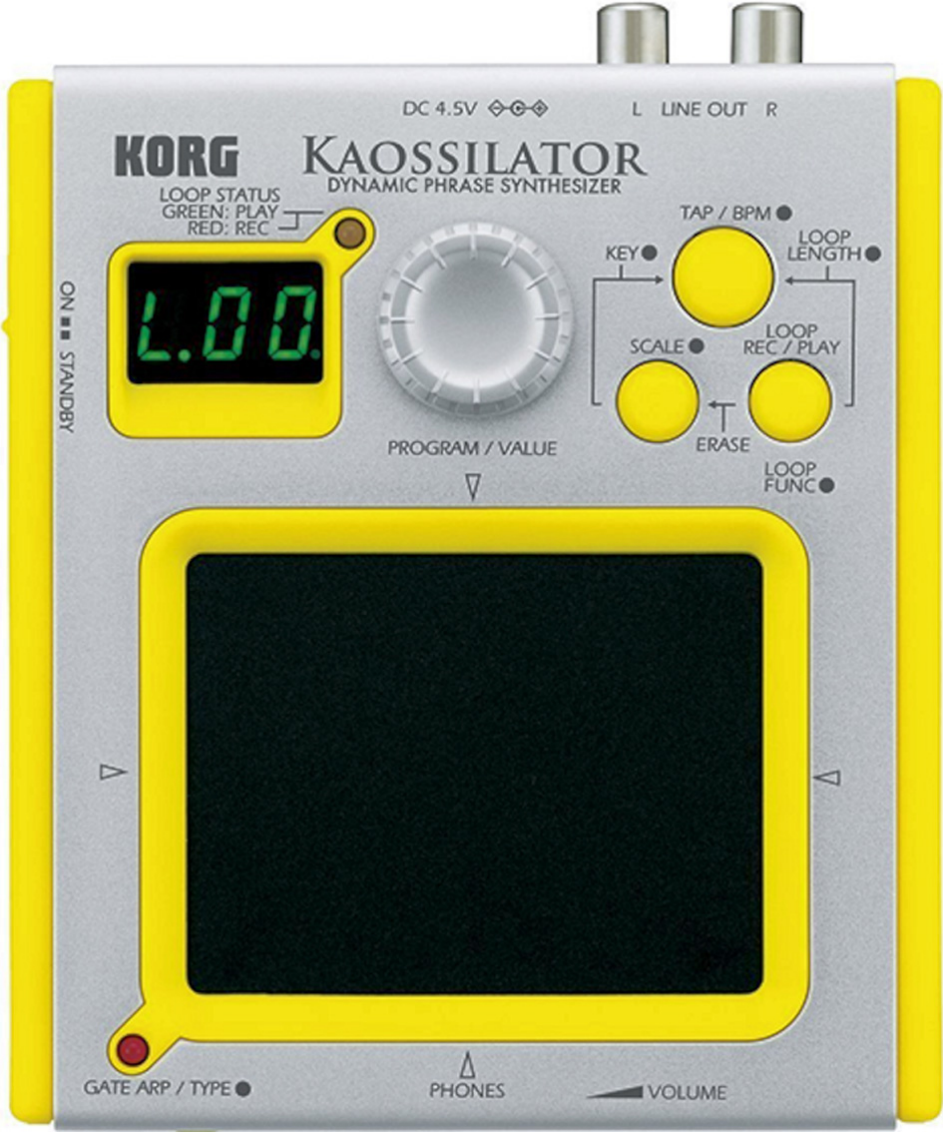
**The synthesizer used**. Participants were asked to create complex sounds by layering sounds into a two bar (8 beats) phrase using a Korg Kaossilator. Sounds are selected via the wheel at the top of the device and synthesized when users touch the black pad in the lower portion of the device. The position of the finger determines the characteristics of the synthetic sounds.

The Korg Kaossilator is an electronic synthesizer controlled from a track pad, like those found on laptop computers, Figure 7. Users can select from 100 different sounds and modify their characteristics via the track pad: the horizontal axis is assigned to note/pitch, while the vertical axis is assigned to parameters such as cutoff, feedback, or modulation depth, allowing the creation of a very wide range of sounds.

A feature we exploit is that music is created by “loop recording”: the device constantly circles through two bars (eight beats) and users can play—and record—layer after layer of music with each repeat. Players may start by recording a rhythm layer of percussive sounds, followed by the gradual addition (overdubbing) of other sounds over the existing layers. Complex music is thus created by repeating this cycle of selecting, playing, and recording sounds. Musicians have to plan their “creations” in multiple additive steps.

The synthesizer is unusual; none of our participants had used it before, but it is designed to be easy to use by novices. All participants were immediately able to produce sounds on it. The use of a small track-pad as an input device means that there are no motion artifacts.

In the target condition subjects were asked to create novel music using the Kaossilator as described above. The memory of the synthesizer was cleared after every four blocks and a regular drum beat (program 90) was preset as a start point for the task.

The CBFV signature recorded in the MCA shows the average haemodynamic modulation over a wide brain area (cf. Figure 1) which includes not only the action planning and musical processing, but also motor and somatosensory activity that is inherent in the manual operation of the synthesizer. To isolate action planning from rhythmic processing and hand movement related activity we asked subjects to tap out a beat, given by a metronome, in the control condition. The target and control condition alternated 20 times during each recording session as described in the general methods section.

#### Participants

Participants were recruited by opportunity sampling or via an experimental participation programme in the School of Psychology at the University of Liverpool and were awarded course credits for their participation in the latter case. We report on data from 26 participants (mean age = 20.52, *SD* = 3.5, 15 female) for the ToL task and on 24 participants (mean age = 20.45, *SD* = 2.05, 16 female) for the music task. All participants also took part in the CWG task described previously. The difference in participant number was due to the strict exclusion criteria we used in the fTCD analysis, which meant that participants with less than 80% of accepted trials during a given task were excluded from the analysis for this task. Data from the majority of participants (21) were available for all three conditions.

For further analysis we categorized our participants as being musically experienced if they had formal musical training for more than 1 year at any point in their life (14). All participants self-reported to be right-handed, all used the right hand to move the token in the ToL task, and all exhibited left lateralized responses during the CWG task. The same subjects performed all three tasks in quasi-random order. The entire recording session took approximately 1 h.

#### fTCD data analysis

**Figure 8:**
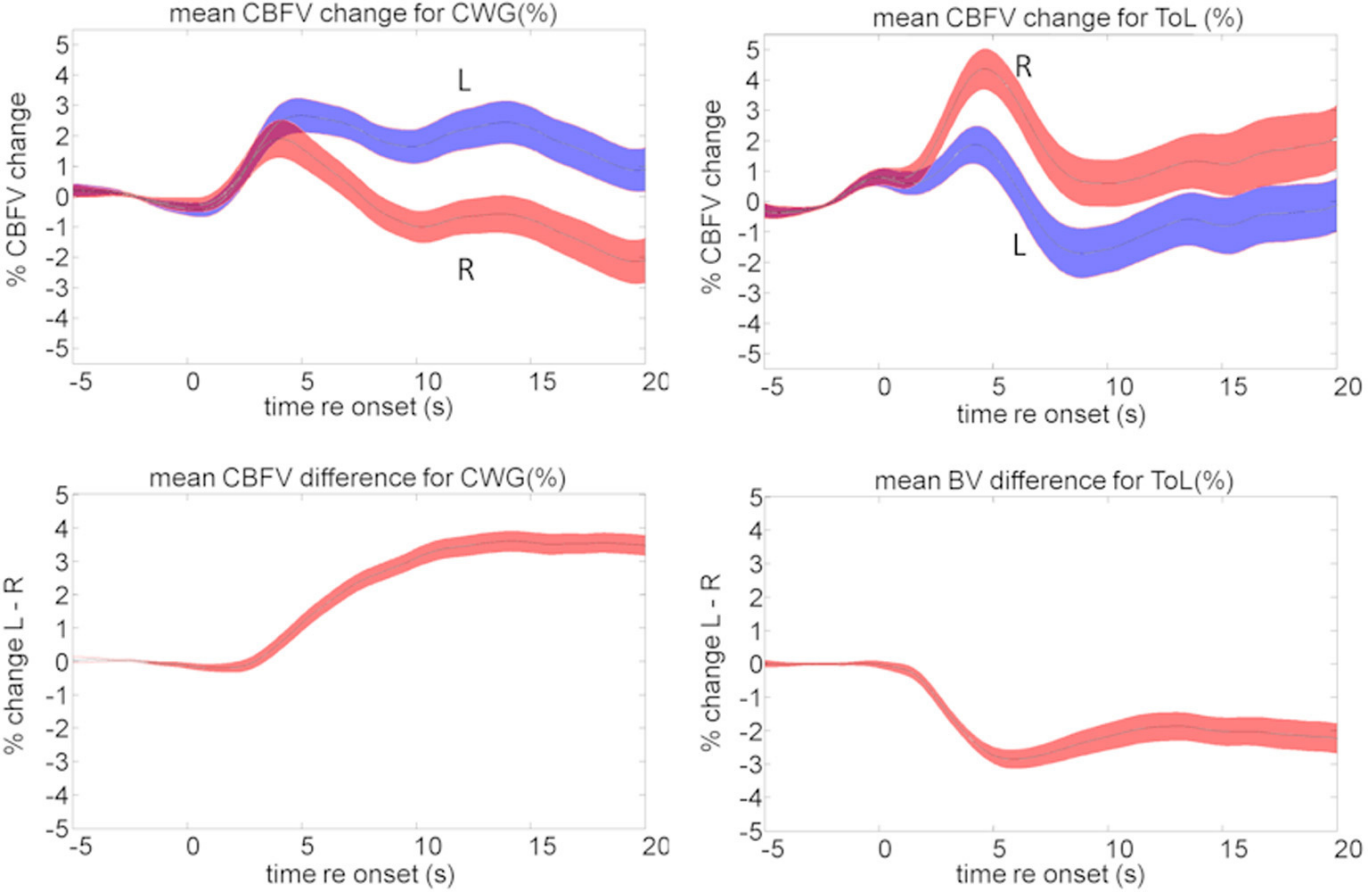
**Mean cerebral blood flow changes (top) and lateralization pattern (bottom) for the cued word generation (CWG, left) and Tower of London (ToL, right) task**. The shaded area defines the standard error over all subjects. The average data shown here hide a significant amount of inter-subject variability that forms the basis of further analysis.

Our fTCD data are consistent with the neuroimaging data discussed above. The language task results in a typical mean activation pattern resulting from a sustained CBFV increase in the left hemisphere while an early transient right lateralized increase in blood-flow is not sustained. The ToL task causes opposite lateralization data: here blood-flow changes are larger for the right hemisphere than for the left, Figure 8.

**Figure 9:**
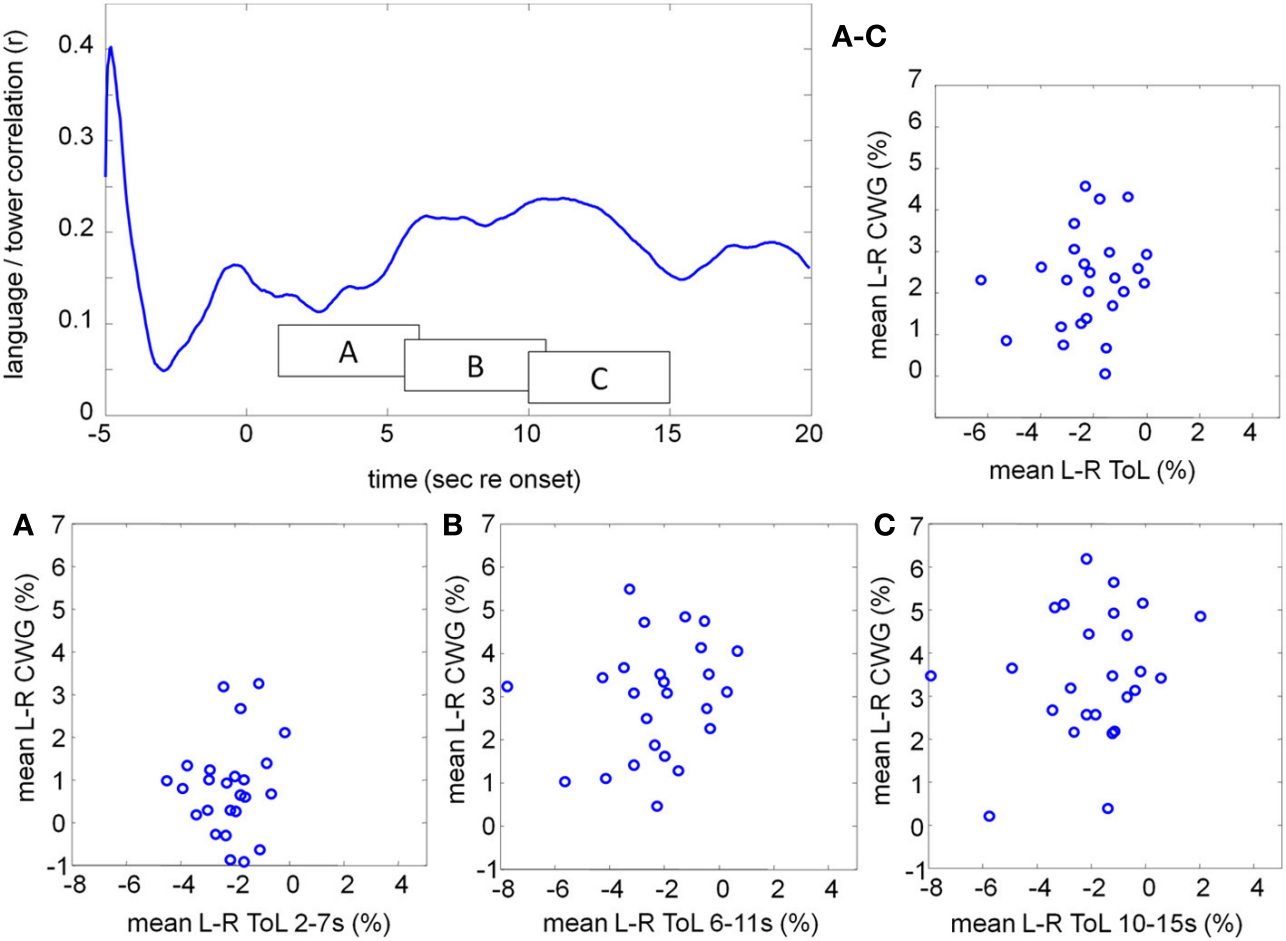
**Correlation between the mean lateralization for CWG and ToL computed across our participant pool using moving windows of 5 s duration (top left)**. The individual lateralization values for the two tasks are never significantly correlated. The average lateralization data in three windows, starting at 2, 6, and 10 s after task onset are shown in boxes A, B, C. The average lateralization for each subject between 2 and 15 s re task onset is shown on the top right **(**labeled **A–C)**.

Figures 9A–C show the mean CBFV changes averaged over 5 s starting at 2, 6, and 10 s after task onset for the CWG and ToL tasks. The correlation of the average CBFV values in moving 5 s windows is shown in the top left graph. The two data sets are never significantly correlated. A peak correlation value (*r* = 0.24, *df* = 24, *p* = 0.25) is seen at 11.2 s after task onset. This dataset extends existing data demonstrating that tasks that draw on different processing networks lead to CBFV patterns that are not correlated.

**Figure 10:**
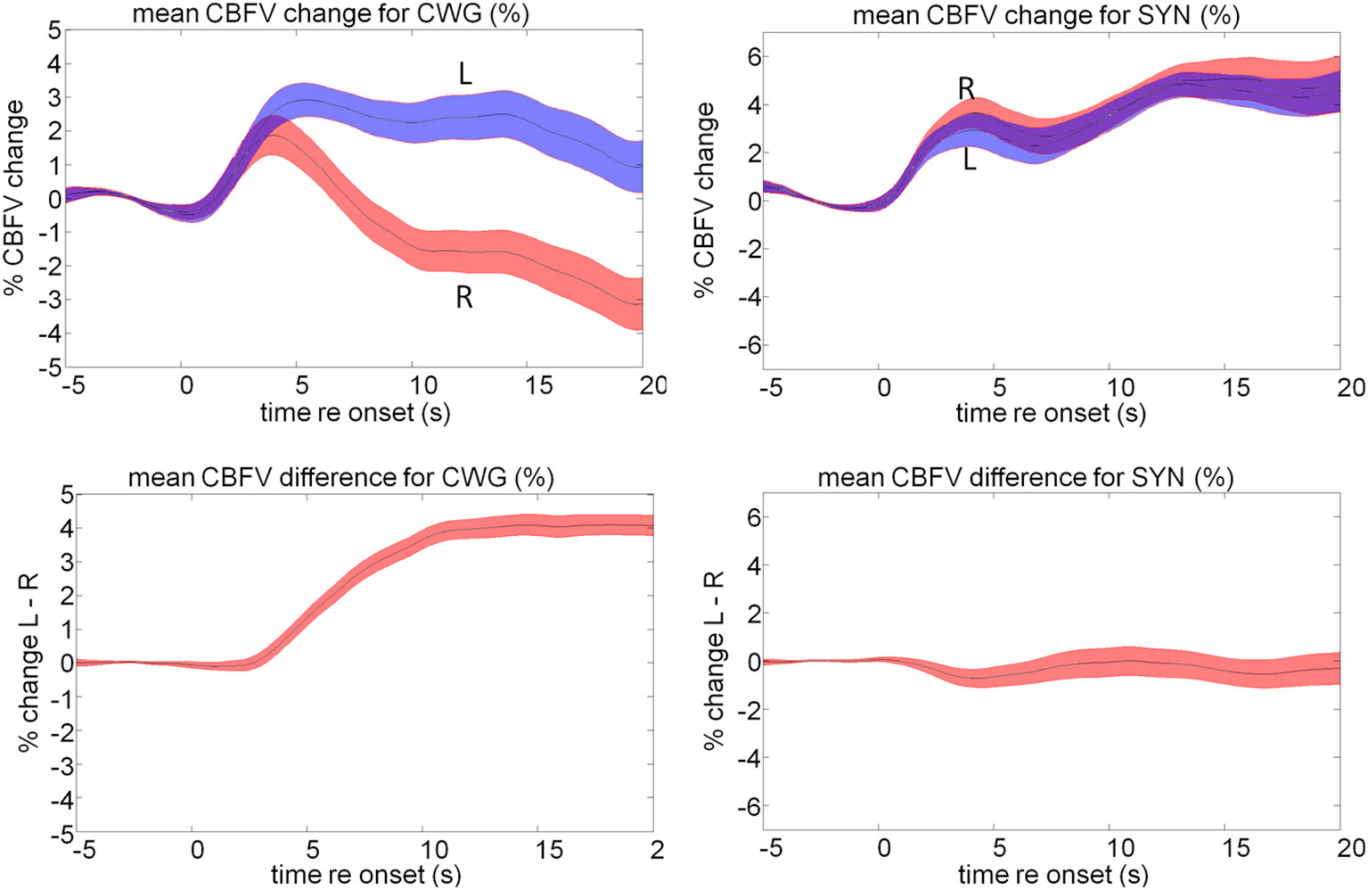
**Mean cerebral blood flow changes (top) and lateralization pattern for the cued word generation (CWG) and music synthesis (SYN) tasks**. The shaded area defines the standard error over all subjects. The average data shown here hide a significant amount of inter-subject variability that forms the basis of further analysis.

**Figure 11:**
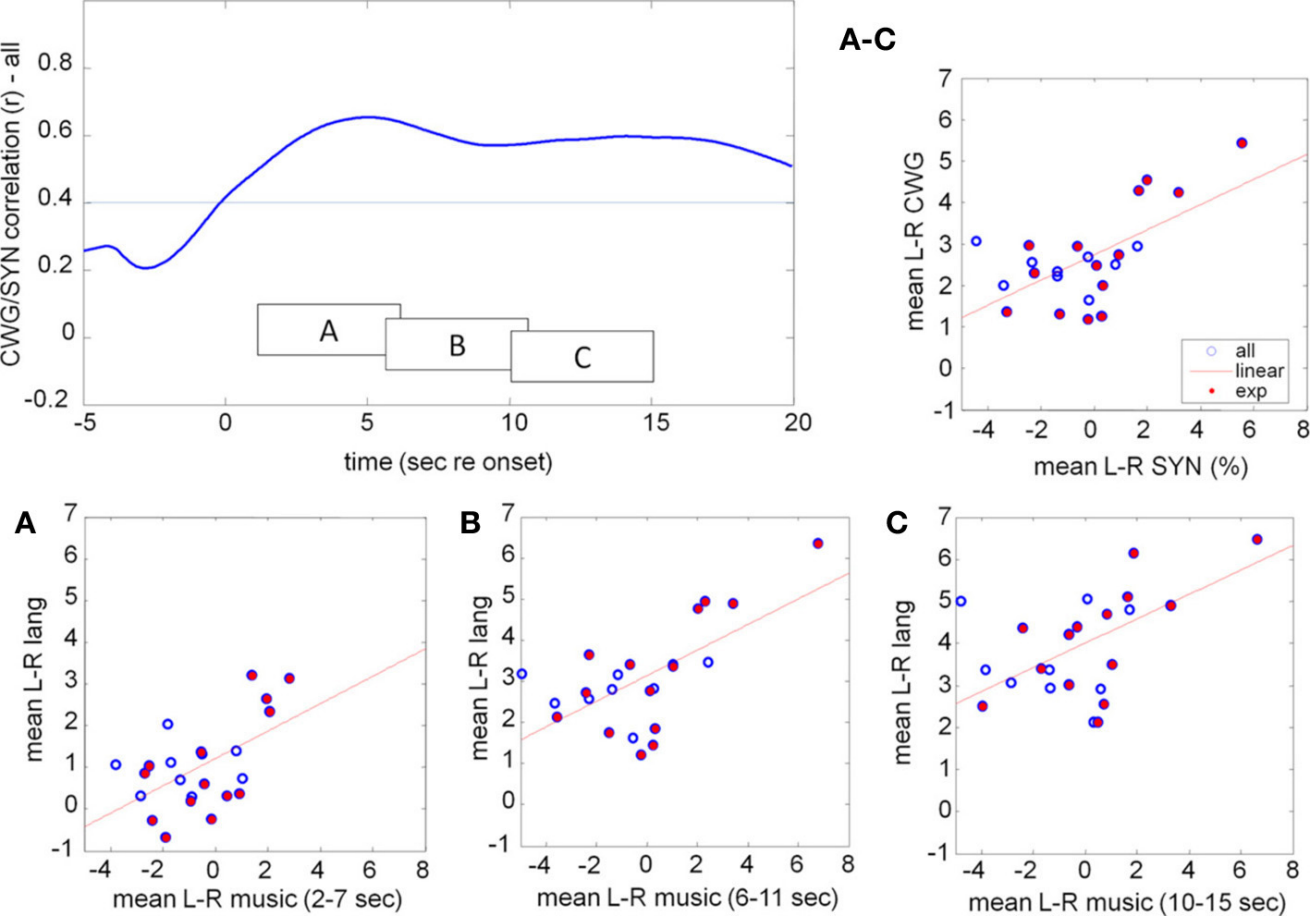
**Correlation between the mean lateralization for CWG and music computed across our participant pool using moving windows of 5 s duration (top left)**. The individual lateralization values for the two tasks are significantly correlated (faint line indicates the *p* = 0.05 threshold) for all analysis windows starting between 0 and 20 s after task onset. The raw data in three windows, starting at 2, 6, and 10 s after task onset are shown in boxes A, B, C. The average lateralization for each subject between 2 and 15 s re task onset is shown on the top right **(**labeled **A–C)**. CBFV for the language task predicts CBFV for music synthesis very well. Participants who received at least 1 year of musical training are marked by the filled red dots.

While the ToL and CWG tasks share few common processing areas in the brain there is a significant body of evidence that links the processing of music and language to common circuits, in particular for trained musicians (Koelsch et al., 2002; Koelsch, 2005; Steinbeis and Koelsch, 2008). The processing of music, however, is not left lateralized, but draws on bilateral cortical areas with a slight bias to the right. As in previous experiments the mean data hide a significant amount of idiosyncratic variation between participants, Figure 10. The range of CBFV data is clearly visible in the mean data, Figure 11. The running cross-task CBFV correlation (Figure 11) shows that the two datasets are significantly correlated over the entire period of task execution. A peak correlation value (*r* = 0.654, *df* = 22, *p* = 0.0007) is seen 5 s after task onset. The data show that the lateralization patterns in all three analysis windows are positively correlated with a slope of approximately 0.33 in all windows.

An important observation is that, while blood-flow is strongly left-lateralized during the CWG task, the music task leads to significant sustained bilateral activity and little overall LI. The correlation analysis, on the other hand, shows highly significant correlations of CBFV for the two tasks. This means that relative LIs are maintained for individuals across the group: those participants who were most strongly left lateralized during the language task also were most left lateralized during music synthesis. Participants who were least left lateralized for language were most right lateralized for music. No participant was right lateralized for language.

The observed lateralization pattern, therefore, should not be interpreted as indicating which hemisphere is used for a particular task; instead it represents the balance of bilateral blood-flow changes. We argue that for this reason, analyses where participants are categorized as being left or right lateralized before further analysis is carried out, are not appropriate.

Evers et al. (1999), on the basis of fTCD data, suggest that musicians and non-musicians have different strategies to lateralize musical stimuli: non-musicians exhibit a delayed but marked right hemisphere lateralization during harmony perception while experienced musicians show enhanced left hemisphere lateralization in an attentive mode of listening. This difference in activation patterns between experienced and inexperienced musicians is also seen in EEG and fMRI studies (Koelsch, 2005; Lahav et al., 2007; Steinbeis and Koelsch, 2008). Bangert et al. (2006) used fMRI to demonstrate that professional pianists showed selective BOLD increases compared to the non-musicians in a distributed cortical network while listening and fingering short piano melodies. The authors argue that a distinct musicianship-specific network, encompassing dorsolateral and inferior frontal cortex as well as superior temporal gyrus, the supramarginal gyrus, and supplementary motor and premotor areas is active in trained musicians. These areas, of course, also define brain areas that are active during speech perception (Meyer et al., 2011, 2013; Beer et al., 2013) and speech production (review: Hickok and Poeppel, 2004, 2007).

If a musicianship-specific network, or specific listening strategies, that share brain areas with the language network exist, then one might expect to see correlated activity for language and music in trained musicians, but not without prior training. Of our 23 participants 14 had received formal musical training for more than 1 year at some point in their life (red filled circles, Figure 11) while another nine had not (open circles).

**Figure 12:**
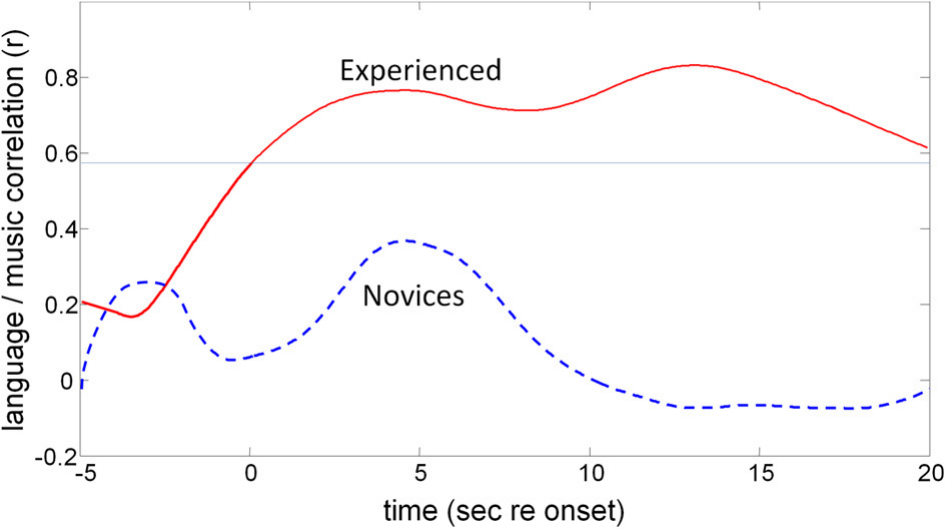
**The effect of previous musical training on CBFV correlation patterns**. The two traces show the results of the moving cross-correlation between CBFV signatures for CWG and music synthesis. The top trace shows the (significant) correlation in blood-flow patterns for experienced musicians while novices (lower trace) do not show significantly correlated activity at any point of execution of the two tasks. The faint horizontal line shows the *p* = 0.05 significance threshold for the experienced participants.

The running correlation (Figure 12) shows that the participants with prior musical training (experienced) show significant correlation between CWG and music synthesis with a peak correlation of *r* = 0.83 (*df* =13, *p* = 0.0001) at around 13 s post task onset while the maximum correlation that is achieved for the musical novices (at 4.4 s, *r* = 0.28, *df* = 8, *p* = 0.46) is not significant.

#### Summary

The fTCD data are consistent with previously published fMRI data, which show that, on average, music perception and execution tasks draw on bilateral networks with a slight bias to the right. The processing network for music nevertheless shares many components with the network used for language processing, so that the observed correlated haemodynamic responses were expected. A number of fMRI studies showed differences in activation patterns for trained and untrained musicians, which presumably resulted from different processing strategies of the two populations for the same signals. These differences are very well represented in the fTCD data which showed that trained musicians exhibit activation patterns that are highly correlated to those seen for language while musical novices show uncorrelated patterns.

Uncorrelated lateralization patterns are also observed throughout the task execution interval when CWG and ToL data are compared. This is consistent with the hypothesis that cognitive tasks that draw on distinct processing networks lead to distinct CBFV patterns while correlated activity is an indication of common processing networks.

## Discussion

Functional TCD recordings, despite the idiosyncrasy of individual blood-flow signatures, are highly replicable. We report on three sets of data for the same (CWG) task that was recorded using different groups of participants and in different environments. The average responses, shown in Figures 2, 8, 10 are very similar: CBFV changes are visible after around 3 s after task onset, then gradually increase over a period of 7–10 s to maximum of 2–3%, which is reached around 13 s after task onset. This makes it possible to compare the recorded data with data from the literature and to evaluate the validity of the recordings.

Individual CBFV lateralization signatures change systematically over time. We therefore argue that any analysis that compares CBFV data across conditions or tasks should take this into account. In experiment 1 we show that the correlation analysis we propose results in similarity measures that are comparable to the conventional LI analysis. We demonstrate that the conventional LI analysis, since it computes peak lateralization values for each task under consideration, may use data from very different phases of the response and therefore may compare data from different time-points. The running correlation avoids this issue. It shows that the relative lateralization patterns for groups of participants are replicable, even at the very early stages of the CBFV response where only small lateralization changes are visible. The analysis therefore provides data that are comparable to the conventional analysis but also provide additional timing information.

For all data reported here the correlation values are obtained for each sample (25 per second) in the response. Our data, consistent with theoretical considerations (Glover, 1999; Jäncke et al., 1999), show that blood-flow changes are relatively slow. For this reason we propose analysis windows of 5 s over which lateralization data are integrated before the correlations are computed. If the time course is evaluated every 2 s, then successive windows have 60% overlap. The correlation measures we report are highly significant for tasks that draw on common brain areas (CWG/CWG; CWG and music) and clearly non-significant for tasks that draw on different areas (CWG and ToL). This means that even an aggressive Bonferroni correction of the results would not change the conclusions; more practical methods such as controlling for false discovery rate (Benjamini and Hochberg, 1995) or using a limited a-priori defined analysis points are also possibilities. The CBFV measure is computed relative to the average values during the baseline period. The inclusion of correlation analysis windows that cover this baseline period provides a useful reality-check for the analysis because in this time window only uncorrelated noise should be measured.

An important consideration when discussing lateralization of cognitive functions is to treat lateralization as a relative dominance of one hemisphere rather than as the exclusive allocation of processing resources to one side of the brain. It means that the relative degree of lateralization across tasks, rather than the location of the dominant side for individual participants, is the relevant measure as we demonstrate in experiment 2.

The conventional LI measure may contribute to the categorical interpretation of fTCD lateralization data because population studies (e.g., Knecht et al., 2000) show a bimodal distribution of LI values. This distribution, which shows few examples near zero, is an inevitable consequence of selecting the maximum values, as Badcock et al. (2012) demonstrate (their Figure 4) by comparing the distribution of mean and maximum lateralization data for the same experiment. It is easy to mistakenly interpret a bimodal distribution of LI values for language as evidence for a bimodal distribution of language lateralization. Instead we show that the mean lateralization data in synchronous time windows can be highly correlated, even if mean lateralization data and the distribution of peak lateralization data (our music/CWG data) might be taken as evidence that the lateralization for the two tasks is different.

FTCD provides data with minimal spatial resolution. In terms of neuroimaging it is clearly not a viable alternative to established techniques such as fMRI and PET. The technique, however, has a number of unique features such as its portability and robustness to participant motion that make it very well suited to complement conventional imaging techniques. The analysis we propose hinges on the assumption that, since there are significant idiosyncratic differences in the haemodynamics for each individual and each brain area, common CBFV patterns for two different tasks are an indication of the invocation of substantially shared brain areas for the processing of both tasks.

The robustness and high degree of replicability of fTCD recordings is well documented (Deppe et al., 1997; Vingerhoets and Stroobant, 2002; Whitehouse et al., 2009) for the conventional LI analysis. A number of studies also show that conventional LIs remain correlated when task difficulty for other tasks is modulated Rosch et al. (2012) and Badcock et al. (2012). Experiment 1 demonstrates that this is also the case for the CWG task and our proposed analysis method. This should not be interpreted as evidence that lateralization and task difficulty are not related, but rather that task-difficulty induced LIs in the CWG task do not affect the correlation significantly.

The main benefit of fTCD is that it can be measured relatively easily, is robust to participant motion and can be used “anywhere.” Uomini and Meyer (2013), who recorded prehistoric stone tool-makers in an open air museum, demonstrate this. This means that one way in which fTCD can complement techniques such as fMRI is by demonstrating that pairs of tasks are correlated. One task could be the target task, the other could be a reference task, which is hypothesized to draw on overlapping brain areas and which can be measured using fTCD and high resolution neuroimaging techniques. CWG, for example serves as a convenient baseline task for language tasks.

Another area where fTCD can complement fMRI is for participant groups where fMRI scanning is problematic. Obvious examples include studies involving children (e.g., Whitehouse et al., 2009) or certain patient groups such as pacemaker users or cochlear implant users.

The fTCD technique and analysis are relatively easy to use and to learn, so that they are very well suited for educational projects. A significant proportion of the data described in this paper were collected as part of university projects or work experience placements for which the relative robustness and safety of the equipment and simplicity of the analysis are invaluable.

### Conflict of interest statement

The authors declare that the research was conducted in the absence of any commercial or financial relationships that could be construed as a potential conflict of interest.
